# Angiogenesis, Inflammation, and Oxidative Stress: Interrelationships in Autoimmune Thyroid Diseases

**DOI:** 10.3390/ijms27062568

**Published:** 2026-03-11

**Authors:** Jelena Djordjevic Milanovic, Vesna Ignjatovic, Katarina Vuleta Nedic, Nevenka Ilic, Marijana Stanojevic Pirkovic, Jelena Nebojsa Terzic, Snezana Zivancevic Simonovic, Nebojsa Zdravkovic, Vladimir Vukomanovic, Nina Urakovic, Vladimir Ignjatovic, Svetlana Kocic, Olgica Mihaljevic

**Affiliations:** 1 Department of Nuclear Medicine and Clinical Oncology, Faculty of Medical Sciences, University of Kragujevac, 34000 Kragujevac, Serbia; jeladj997@gmail.com (J.D.M.); vesnaivladaignjatovic@gmail.com (V.I.); kvuleta87@gmail.com (K.V.N.); vukomanovic@gmail.com (V.V.); 2 University Clinical Center Kragujevac, 34000 Kragujevac, Serbia; marijanas14@gmail.com (M.S.P.); n.urakovic@gmail.com (N.U.); vladaig@yahoo.com (V.I.); 3 Department of Allergology and Immunology, Institute for Public Health Kragujevac, 34000 Kragujevac, Serbia; ilic.nena.nevenka@gmail.com; 4 Department of Biochemistry, Faculty of Medical Sciences, University of Kragujevac, 34000 Kragujevac, Serbia; 5 Department of Pharmacy, Faculty of Medical Sciences, University of Kragujevac, 34000 Kragujevac, Serbia; jelena.terzic@fmn.kg.ac.rs; 6 Center of Excellence for the Study of Redox Balance in Cardiovascular and Metabolic Disorders, University of Kragujevac, 34000 Kragujevac, Serbia; 7 Department of Pathophysiology, Faculty of Medical Sciences, University of Kragujevac, 34000 Kragujevac, Serbia; snezana@fmn.kg.ac.rs; 8 Department of Medical Statistics and Informatics, Faculty of Medical Sciences, University of Kragujevac, 34000 Kragujevac, Serbia; nzdravkovic@fmn.kg.ac.rs; 9 Department of Internal Medicine, Faculty of Medical Sciences, University of Kragujevac, 34000 Kragujevac, Serbia; 10 Department of Radiology, Clinical Hospital Center, Zemun, 11070 Belgrade, Serbia; lanakocic@gmail.com

**Keywords:** autoimmune thyroid diseases, angiogenesis, inflammation, oxidative stress

## Abstract

Autoimmune thyroid diseases (AITD) are based on reactivity to thyroid self-antigens, resulting in varying degrees of persistent inflammation and glandular hyperplasia. The aim of this study was to investigate the interplay between angiogenesis, inflammation, and oxidative stress in patients with AITD. The study included patients with AITD, divided into a group with Hashimoto’s thyroiditis (HT) and a group with Graves’ disease (GD), as well as healthy controls. The results showed that subjects with GD had significantly higher concentrations of angiopoietin-2 (Ang-2) compared to those with HT and the healthy controls (*p* < 0.001). Inflammatory parameters (C-reactive protein (CRP), the systemic inflammatory immune response index (SII), and the CRP/albumin ratio (CRP/alb)) were higher in both AITD groups (*p* < 0.001). Oxidative stress parameters were more pronounced in AITD, while the activity of antioxidant enzymes was reduced. Ang-2 positively correlated with H_2_O_2_ (r = 0.394, *p* = 0.006) and NO (r = 0.519, *p* = 0.001) in HT, as well as with O_2_^−^ (r = 0.232, *p* = 0.009) and TBARS (r = 0.190, *p* = 0.038) in GD, while in GD it showed a negative correlation with SOD (r = −0.426, *p* = 0.012) and CAT (r = −0.534, *p* = 0.008). Thus, angiogenesis, inflammation, and oxidative stress are interconnected processes in AITD, which may have significance for further understanding of the disease and the development of therapeutic approaches.

## 1. Introduction

Autoimmune thyroid diseases (AITD) are organ-specific autoimmune diseases, most commonly including Hashimoto’s thyroiditis (HT) and Graves’ disease (GD) [1]. HT is characterized by marked inflammatory changes, dense lymphocytic infiltration, and progressive destruction of thyroid tissue, while GD is characterized by thyroid hyperplasia and increased neovascularization, usually accompanied by a less pronounced lymphocytic infiltrate [2,3]. In AITD, endothelial cells have been reported to regulate vascular permeability and leukocyte extravasation in the thyroid gland, contributing to inflammation and angiogenesis [4].

Inflammation and angiogenesis are closely linked processes, particularly under pathological conditions. Angiogenesis, defined as the generation of new blood vessels from the existing vascular network, is controlled by a dynamic and tightly regulated interplay between pro-angiogenic and anti-angiogenic mediators [5]. The formation of new blood vessels supports the sustained infiltration of inflammatory cells, which secrete pro-angiogenic cytokines, chemokines, and growth factors, thereby amplifying and perpetuating the angiogenic process [6].

In recent years, considerable attention has been devoted to identifying and characterizing reliable biomarkers that reflect angiogenic activity in the thyroid gland [7]. Although a large number of factors are important in this complex process, the angiopoietin (Ang) family appears to play a key role in the maturation, survival, and remodeling of the vascular bed of thyroid tissue [8]. In an immunohistochemical study, the expression of angiopoietin-2 (Ang-2) and its receptors was determined not only in follicular and endothelial cells of healthy thyroid tissue, but also in benign and malignant lesions of the thyroid gland [9]. However, studies on the pathophysiological role of Ang-2 in AITD are quite scarce. Several researchers have noted significantly higher concentrations of Ang-2 in these patients, as well as the association of this biomarker with levels of free triiodothyronine (fT3) and free thyroxine (fT4) [10,11]. The results of their studies point to Ang-2 as a mediator of angiogenesis and vascular network remodeling, but the exact role of this biomarker in AITD patients remains insufficiently investigated.

In addition, thyroid diseases are increasingly associated with oxidative stress. An imbalance between oxidants and antioxidants has been observed in AITD patients at different stages of the disease [12,13]. Changes in thyroid hormone concentrations can cause changes in the activity of the mitochondrial respiratory chain, ultimately leading to increased generation of free radicals (ROS) [2]. In a patient with GD, the overproduction of thyroid hormones causes oxidative stress through increased production of ROS; this is in contrast to HT, where the redox imbalance can be attributed to an ineffective antioxidant system [14]. Interestingly, an imbalance in the production of ROS leads to increased synthesis of inflammatory mediators, which ultimately activates angiogenesis [15].

The aim of our study was to analyze serum concentrations of Ang-2 in patients with AITD, as well as to examine the association of Ang-2 with markers of inflammation, oxidative stress, antioxidant protection, and selected laboratory parameters.

## 2. Results

### 2.1. Demographic and Clinical Characteristics of the Study Population

Three groups of subjects were analyzed in the study: 97 subjects diagnosed with HT (16.1% men and 83.9% women), 86 subjects diagnosed with GD (22.7% men and 77.3% women), and 76 healthy subjects (35.5% men and 64.5% women). The average age of subjects diagnosed with HT was 50.05 ± 16.83 years (range 18–82 years) and the average age of subjects diagnosed with GD was 54.58 ± 16.44 (range 21–89 years), while the average age of subjects from the control group was 42.54 ± 16.03 (range 18–85 years). There was no statistically significant difference in gender distribution (χ^2^ = 1.522, *p* = 0.562) and average age (*p* = 0.215) of the respondents among the analyzed groups.

In the group of subjects with HT, 29 (29.9%) gave a positive answer about cigarette consumption, while the remaining 68 (70.1%) declared themselves non-smokers. Among the subjects with GD, there were 27 (31.4%) smokers and 59 (68.6%) non-smokers, as well as 17 (22.4%) smokers and 59 (77.6%) non-smokers among the healthy controls. Analysis of smoking frequency excluded the existence of significant differences among the subjects of the three study groups (χ^2^ = 1.534, *p* = 0.465).

**Figure 1:**
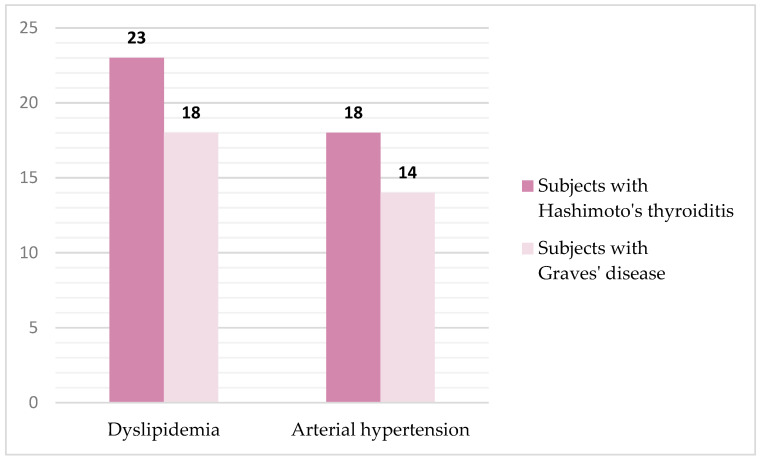
Representation and type of comorbidity in the population of subjects with autoimmune thyroid diseases.

The presence of comorbidity was confirmed in 73 (27.8%) subjects, namely: 41 subjects with HT and 32 subjects with GD. Representation and type of comorbidity in the population of subjects with AITD are shown in Figure 1. Statistical analysis of the collected data showed that there was no significant difference in the frequency of comorbidity between the two groups of subjects with AITD.

The parameters of the thyroid function of the studied population are shown in Table 1.

### 2.2. Serum Concentration of Angiopoietin-2 in the Study Population

**Figure 2:**
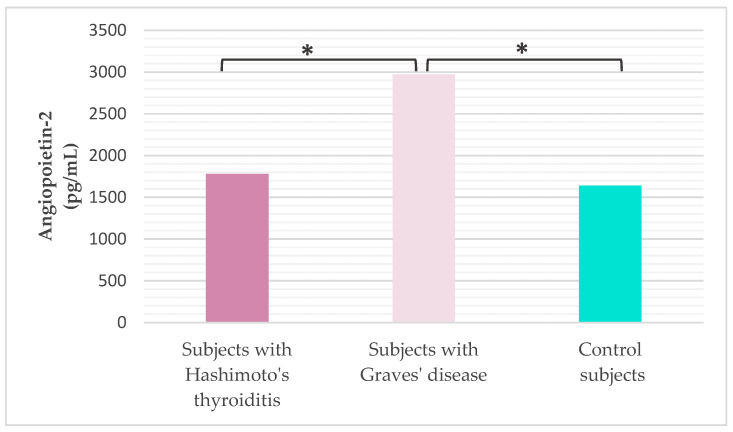
Concentration of serum angiopoietin-2 in the study population (* Tukey HSD post hoc test, *p* ≤ 0.05 was considered statistically significant).

Serum Ang-2 concentration was analyzed in all subjects (Figure 2). It was found that subjects with GD have a statistically significantly higher serum concentration of Ang-2 compared to subjects diagnosed with HT (Tukey HSD post hoc test, *p* < 0.001) and healthy controls (Tukey HSD post hoc test, *p* < 0.001).

### 2.3. Hematological, Biochemical and Inflammation Parameters in the Study Population

The mean concentration values of selected hematological and biochemical parameters in the studied population are shown in Table 2.

The number of leukocytes, neutrophils, lymphocytes, platelets, serum iron concentration, and the values of platelet indices mean platelet volume (MPV) and platelet distribution width (PDW) were significantly lower in the control group of subjects compared to subjects with HT (Mann–Whitney U test, *p*_leukocytes_ < 0.001; Mann–Whitney U test, *p*_neutrophils_ < 0.001; Tukey HSD post hoc, *p*_lymphocytes_ = 0.008, Mann–Whitney U test, *p*_platelets_ = 0.001;Tukey HSD post hoc *p*_iron_ = 0.008, Mann–Whitney U test; *p*_MPV_ = 0.012; Tukey HSD post hoc, *p_PDW_* = 0.021) and subjects with GD (Mann–Whitney U test, *p*_leukocytes_ < 0.001; Mann–Whitney U test, *p*_neutrophils_ = 0.002; Tukey HSD post hoc, *p*_lymphocytes_ = 0.049, Mann–Whitney U test, *p*_platelets_ = 0.001;Tukey HSD post hoc *p*_iron_ = 0.023, Mann–Whitney U test; *p*_MPV_ = 0.006; Tukey HSD post hoc, *p*_PDW_ = 0.025), while we excluded statistically significant differences between the two groups of subjects with AITD (Mann–Whitney U test, *p*_leukocytes_ = 0.909; Mann–Whitney U test, *p*_neutrophils_ = 0.892; Tukey HSD post hoc, *p*_lymphocytes_ = 0.781, Mann–Whitney U test, *p*_platelets_ = 0.411;Tukey HSD post hoc *p*_iron_ = 0.884, Mann–Whitney U test; *p*_MPV_ = 0.112; Tukey HSD post hoc, *p*_PDW_ = 0.213).

Regarding the biochemical parameters, a statistically significantly higher concentration of urea (*p* = 0.003) and uric acid (*p* < 0.001) was found in subjects diagnosed with GD compared to the rest of the study population. Vitamin D concentration was similar between the two groups of subjects with AITD (Mann–Whitney U test, *p* = 0.643), but significantly lower compared to healthy controls (Mann–Whitney U test, *p* = 0.015 for subjects with HT; *p* = 0.003 for subjects with GD).

When it comes to inflammation parameters (Table 3), it was found that the concentration of C-reactive protein (CRP), as well as the value of the systemic inflammatory immune response index (SII) and the ratio of CRP to albumin (CRP/alb), were significantly higher in subjects with HT (Tukey HSD post hoc, *p*_CRP_ = 0.006; Tukey HSD post hoc, *p*_SII_ = 0.001; Tukey HSD post hoc, *p*_CRP/alb_ = 0.035) and subjects with GD (Tukey HSD post hoc, *p*_CRP_ = 0.001; Tukey HSD post hoc, *p*_SII_ = 0.048; Tukey HSD post hoc, *p*_CRP/alb_ < 0.001) compared to the control group. A significantly higher concentration of interleukin 6 (IL-6) was recorded in subjects with GD compared to the group of subjects with HT (Tukey HSD post hoc, *p* = 0.002) and the group of healthy subjects (Tukey HSD post hoc, *p* < 0.001).

### 2.4. Parameters of Oxidative Stress and Antioxidant Protection in the Study Population

**Figure 3:**
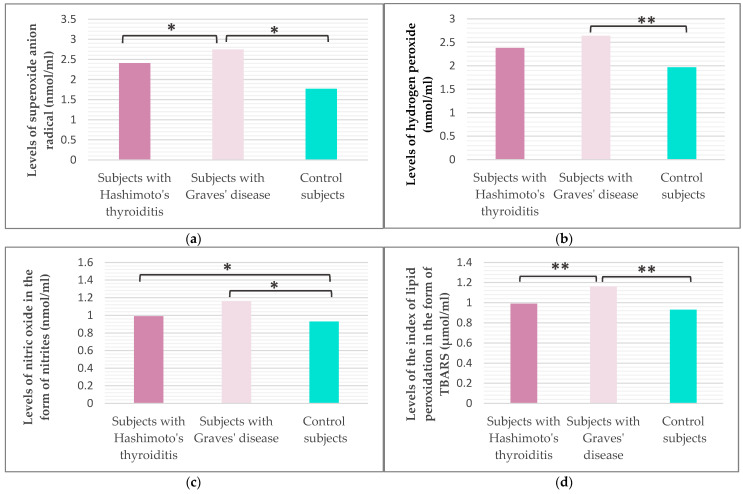
Values of superoxide anion radical (**a**), hydrogen peroxide (**b**), nitric oxide in the form of nitrite (**c**), and lipid peroxidation index in the form of TBARS (**d**) in the study population (* Tukey HSD post hoc, *p* ≤ 0.05 was considered statistically significant.; ** Mann–Whitney test with Bonferroni correction, *p* < 0.017 was considered statistically significant).

**Figure 4:**
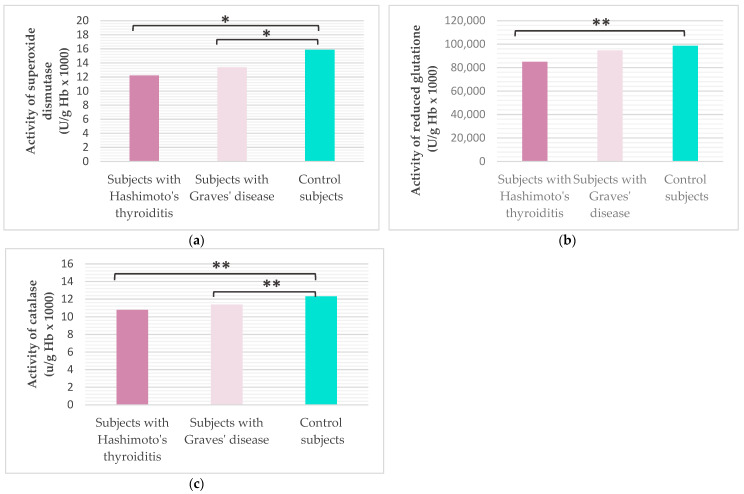
Values of superoxide dismutase (**a**), catalase (**b**) and reduced glutathione (**c**) in the study population (* Tukey HSD post hoc, *p* ≤ 0.05 was considered statistically significant.; ** Mann–Whitney test with Bonferroni correction, *p* < 0.017 was considered statistically significant).

Our study included the analysis of the parameters of oxidative stress (Figure 3) and antioxidant protection (Figure 4) in the studied population. It was shown that subjects with GD have statistically significantly higher values of superoxide anion radicals (O_2_^−^) and lipid peroxidation with thiobarbituric acid (TBARS) compared to subjects with HT (Tukey HSD post hoc, *p*_O2_ = 0.028; Mann–Whitney U test, *p*_TBARS_ = 0.013) and healthy controls (Tukey HSD post hoc, *p*_O2_ < 0.001; Mann–Whitney U test, *p*_TBARS_ < 0.001). No statistically significant difference in hydrogen peroxide (H_2_O_2_) values was shown between the two groups of subjects with AITD (Mann–Whitney U test, *p* = 0.207), but the average value of H_2_O_2_ was significantly higher in patients with GD compared to healthy controls (Mann–Whitney U test, *p* = 0.006). The value of nitric oxide (NO) was significantly higher in subjects with HT (Tukey HSD post hoc, *p* = 0.020) and in subjects with GD (Tukey HSD post hoc, *p* < 0.001) compared to the control group of healthy subjects.

Analysis of antioxidant protection enzymes revealed a statistically significantly lower activity of superoxide dismutase (SOD) and catalase (CAT) among subjects with HT (Tukey HSD post hoc, *p*_SOD_ = 0.047; Mann–Whitney U test, *p*_CAT_ = 0.002) and subjects with GD (Tukey HSD post hoc, *p*_SOD_ = 0.045; Mann–Whitney U test, *p*_CAT_ = 0.015) compared to healthy controls. Glutathione (GSH) activity was significantly lower in subjects with HT compared to healthy controls (Mann–Whitney U test, *p* < 0.001), while no significant difference was shown between groups of subjects with AITD (Mann–Whitney U test, *p* = 0.829).

### 2.5. Association of Parameters of Thyroid Function, Hematological, Biochemical, Parameters of Inflammation and Oxidative Stress with Serum Concentration of Angiopoietin-2 in the Studied Population

**Figure 5:**
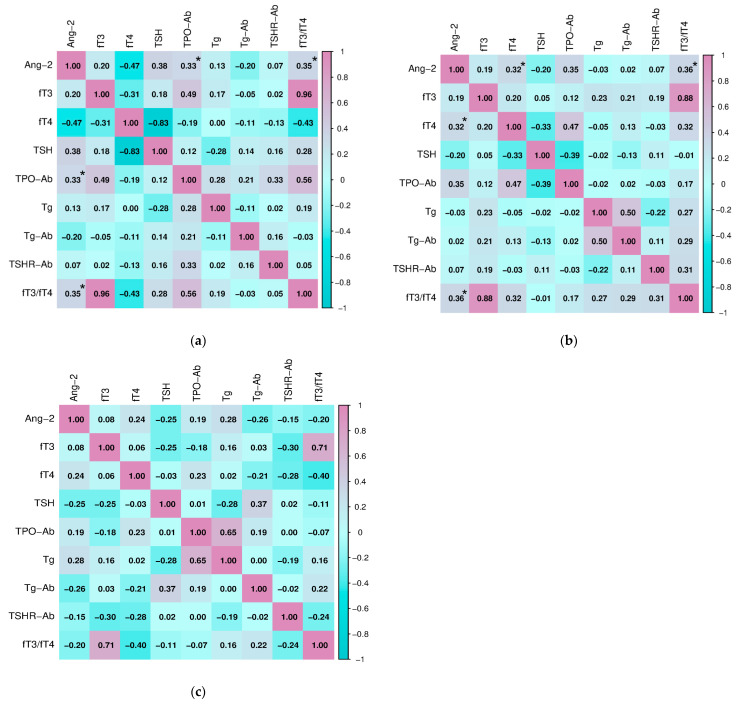
Correlation of angiopoietin-2 with thyroid function parameters in (**a**) subjects with Hashimoto’s thyroiditis, (**b**) subjects with Graves’ disease, and (**c**) healthy control (* *p* ≤ 0.05 was considered statistically significant).

After analyzing the differences in the values of the selected parameters between the three groups of subjects, we examined the potential connection, i.e., correlated the serum concentration of Ang-2 with the analyzed parameters of thyroid function, hematological, biochemical, inflammation, and oxidative stress parameters in the study population. Statistically significant relationships between Ang-2 and parameters of thyroid function in two groups of subjects with AITD were shown (Figure 5). Serum Ang-2 concentration positively correlated with thyroid peroxidase antibodies (TPO-Ab) (Spearman, r = 0.330, *p* = 0.040) and the fT3/fT4 ratio (Pearson, r = 0.349, *p* = 0.044) in subjects with HT. Also, in the group of subjects with GD, Ang-2 significantly correlated with the fT3/fT4 ratio (Spearman, r = 0.359, *p* = 0.009), as well as with ft4 concentration (Spearman, r = 0.320, *p* = 0.046). There were no significant associations in the healthy subjects.

**Figure 6:**
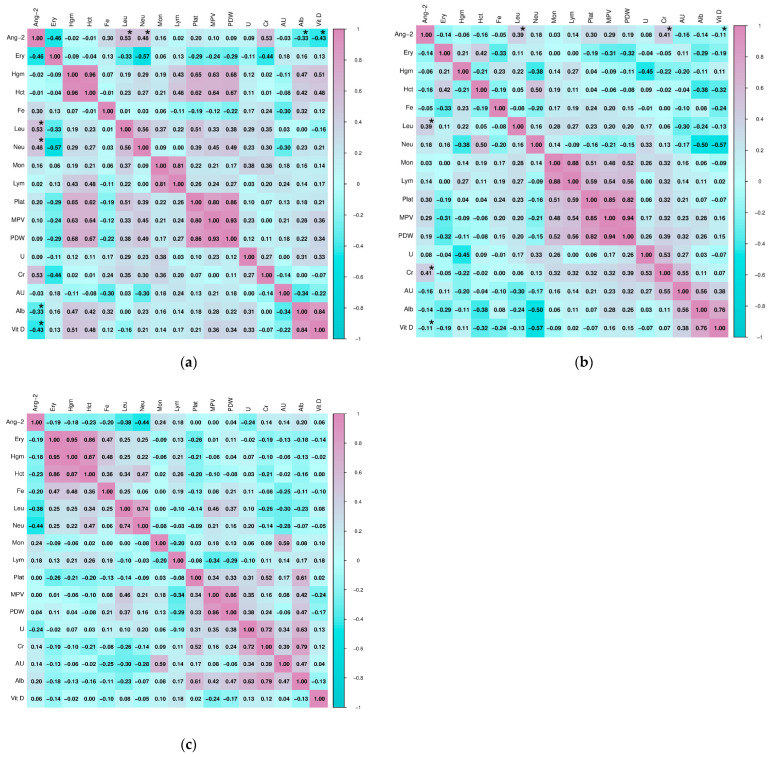
Correlation of angiopoietin-2 with hematological and biochemical parameters in (**a**) subjects with Hashimoto’s thyroiditis, (**b**) subjects with Graves’ disease, and (**c**) healthy controls (* *p* ≤ 0.05 was considered statistically significant).

Testing the relationship of Ang-2 with selected hematological and biochemical parameters in subjects with HT revealed a statistically significant positive correlation with the absolute number of leukocytes (Spearman, r = 0.527, *p* = 0.002) and neutrophils (Pearson, r = 0.482, *p* = 0.005), as well as significant negative correlations with the concentration of albumin (Spearman, r = −0.334, *p* = 0.001) and vitamin D (Spearman, r = −0.428, *p* = 0.023). In subjects with GD, a statistically significant correlation was shown for the relationship of Ang-2 with the number of leukocytes (Pearson, r = 0.388, *p* = 0.021) and creatinine concentration (Pearson, r = 410, *p* = 0.047), as well as a significant negative correlation with the concentration of vitamin D (Spearman, r = −0.113, *p* = 0.025). In the group of healthy subjects, there were no statistically significant correlations of hematological and biochemical parameters with Ang-2. The association of other hematological and biochemical parameters with Ang-2 in the study population is shown in Figure 6.

**Figure 7:**
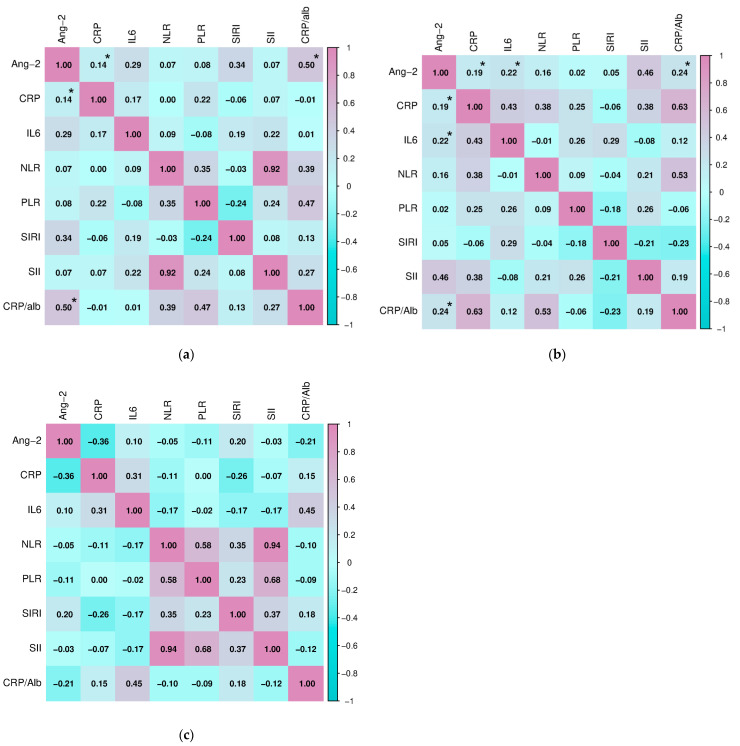
Correlation of angiopoietin-2 with inflammation parameters in (**a**) subjects with Hashimoto’s thyroiditis, (**b**) subjects with Graves’ disease, and (**c**) healthy controls (* *p* ≤ 0.05 was considered statistically significant).

During further research, we examined the association of serum Ang-2 concentration with parameters of inflammation in the study population (Figure 7). There was a significant positive relationship between the concentration of Ang-2 and CRP (Pearson, r = 0.144, *p* = 0.043) and the value of the CRP/alb index (Pearson, r = 0.502, *p* = 0.011) in subjects with HT. Similarly, Ang-2 correlated with the concentration of CRP (Pearson, r = 0.188, *p* = 0.041) and the value of the CRP/alb index (Pearson, 0.239, *p* = 0.008), but also with the concentration of IL-6 (Pearson, r = 0.224, *p* = 0.029) in the group of subjects diagnosed with GD. There were no significant associations in the healthy subjects.

**Figure 8:**
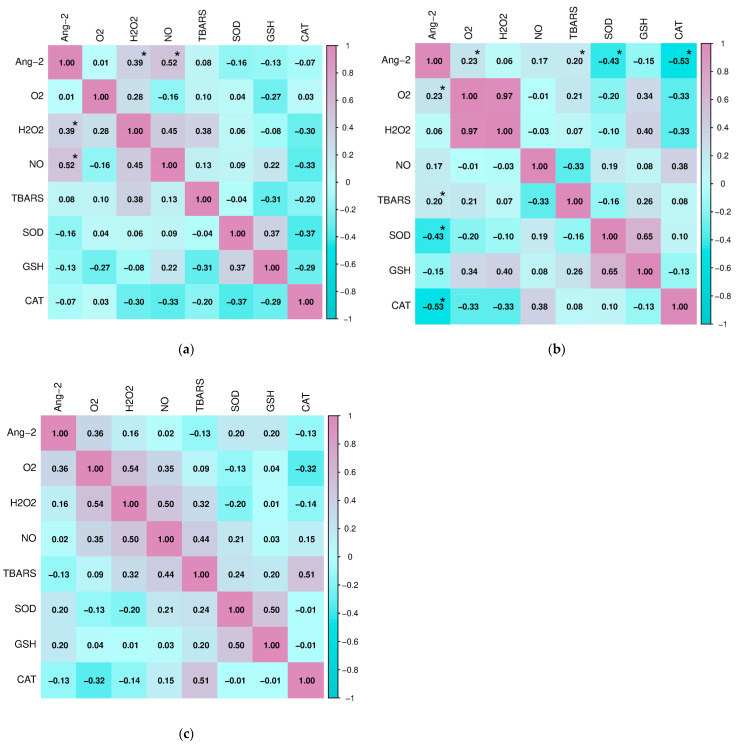
Correlation of angiopoietin-2 with parameters of oxidative stress and antioxidative protection in (**a**) subjects with Hashimoto’s thyroiditis, (**b**) subjects with Graves’ disease, and (**c**) healthy controls (* *p* ≤ 0.05 was considered statistically significant).

Finally, we analyzed the relationship of Ang-2 with parameters of oxidative stress and antioxidant protection in all three groups of subjects (Figure 8). In the group of subjects with HT, a positive correlation of Ang-2 with H_2_O_2_ (Spearman, r = 0.394, *p* = 0.006) and NO (Pearson, r = 0.519, *p* = 0.001) was confirmed, while in subjects with GD, angiopoietin-2 positively correlated with O_2_^−^ (Pearson, r = 0.232, *p* = 0.009) and TBARS (Pearson, r = 0.190, *p* = 0.038). Also, in the group of subjects with GD, there was a significant negative correlation of Ang-2 concentration with the activity of the antioxidant enzymes SOD (Pearson, r = −0.426, *p* = 0.012) and CAT (Pearson, r = −0.534, *p* = 0.008). No statistically significant association was found between Ang-2 and parameters of oxidative stress and antioxidant protection in the control group.

## 3. Discussion

The aim of this study was to evaluate the serum concentration of Ang-2, parameters of inflammation, oxidative stress, and antioxidant protection in patients with AITD, who were divided into subjects with HT and subjects with GD. In addition, we clarified the relationship between Ang-2 concentration and certain laboratory parameters, as well as the relationship of Ang-2 with parameters of inflammation, oxidative stress, and antioxidant protection. To our knowledge, this is the first study to analyze the relationship between angiogenesis, inflammation, and oxidative stress in AITD patients.

Previous studies have suggested that Ang-2 may play a significant role in the pathogenesis of various inflammatory and autoimmune diseases [16,17]. The presence of Ang-2 has been noted in inflammatory conditions caused by autoimmune diseases such as rheumatoid arthritis, systemic lupus erythematosus, vasculitis, and psoriasis [18]. However, the number of studies investigating this biomarker in AITD is scarce. Figueroa-Vega et al. have found in their work that the serum of patients with GD shows a “proangiogenic” profile with increased levels of Ang-2 and Tie-2. Also, the researchers reported that there was a close relationship between Ang-2, Tie-2, fT4, and thyroid-stimulating hormone receptor (TSHR-Ab) levels in GD patients [11]. In accordance with their results, our research indicates that subjects with GD have significantly higher concentrations of Ang-2 compared to subjects with HT and healthy controls. The higher level of Ang-2 in subjects with GD could probably be explained by the more intense angiogenic activity and metabolic demands of the hyperthyroid state. In addition, in subjects with GD, we found a positive correlation between Ang-2 with fT4 concentration and the fT3/fT4 ratio, while in subjects with HT, a positive correlation of Ang-2 with TPO-Ab was also recorded. These data show that both thyroid function and autoantibody titers can potentially have an impact on angiogenic signaling pathways. In contrast, although thyroglobulin (Tg) is considered an important indicator of follicular cells integrity and autoimmune process activity [19], our study did not find an association between Tg and Ang-2. These findings suggest that the processes leading to Tg release due to tissue damage may not be directly related to angiogenic signaling pathways in AITD.

Growing evidence suggests that angiopoietins (Ang) stimulate the expression of proinflammatory genes, including those encoding different chemokines and tumor necrosis factor alpha (TNF-α). Earlier studies have shown that Ang-2 may potentiate inflammatory signaling by enhancing endothelial cell responsiveness to TNF-α and vascular endothelial growth factor (VEGF), supporting the concept of a shared regulatory pathway linking inflammation and angiogenesis [20]. Additionally, the angiopoietin system has been shown to promote chemotaxis of peripheral blood mononuclear cells, facilitating their recruitment to tissues and contributing to the amplification of inflammatory processes [21]. In both groups of our subjects with AITD, higher values of formed elements of white blood lineage (absolute number of leukocytes, neutrophils, and lymphocytes) and inflammatory markers (CRP, SII, CRP/alb, and IL-6) were recorded compared to healthy controls. The absolute number of leukocytes, the concentration of CRP, IL-6, as well as the value of the CRP/alb ratio, showed a positive relationship with the concentration of Ang-2 in patients with AITD, which confirms that angiogenesis and inflammation are two correlated conditions. Similarly, in a study conducted by a group of German researchers, serum Ang-2 levels were positively associated with inflammatory markers (CRP, white blood cell count, and IL-6) [22]. Research conducted on animal models also indicates that Ang-2 can be considered an important parameter for the initiation of an inflammatory response, as mice deficient in Ang-2 were unable to initiate an inflammatory response during infection [23]. In addition, it is interesting that Ang-2 showed a significant negative association with vitamin D concentration in both subjects with HT and subjects with GD. Vitamin D is known for its immunomodulatory and anti-inflammatory effects, as well as for its potential role in the regulation of angiogenesis [24]. The results of recent studies indicate that vitamin D deficiency may contribute to increased angiogenic and inflammatory activity, while vitamin D supplementation leads to a decrease in angiogenesis through the suppression of the activation of proliferation, migration, and germination of endothelial cells [25].

Recently, the concept of the interdependence of angiogenesis and inflammation has been extended to include oxidative stress [26]. Oxidative stress can be caused by excessive generation of ROS but also by an ineffective antioxidant system, which leads to molecular damage. Since ROS are necessary in the initial stages of thyroid hormone production, the thyroid gland is particularly exposed to oxidative stress [27]. Moreover, in mouse models, oxidative damage was observed much more frequently in the thyroid gland than in other organs [28]. Thus, in the thyroid, antioxidant defense enzymes must effectively regulate the production and removal of ROS [29], otherwise oxidative stress may impair autotolerance and consequently lead to thyroid autoimmune dysfunction [30]. Our study noted that subjects with AITD have high levels of all analyzed parameters of oxidative stress compared to healthy subjects. A significant increase in O_2_^−^ and TBARS concentrations was recorded in subjects with GD compared to both healthy controls and subjects with HT. When it comes to antioxidant protection, subjects with AITD showed lower activity of antioxidant protection enzymes. All analyzed parameters of antioxidant protection (SOD, CAT, and GSH) had reduced activity in subjects with HT compared to healthy controls. These results are consistent with previous research indicating that hypothyroidism contributes to oxidative stress through an ineffective antioxidant defense system; this is in contrast to hyperthyroidism, where increased ROS production promotes oxidative stress and oxidative damage to thyroid cells [31]. Excessive production of ROS can cause the production of pro-inflammatory cytokines, and the available literature shows that inflammation and oxidative stress can further lead to the activation of angiogenesis [32]. Bearing this in mind, we also evaluated the relationship of prooxidative/antioxidant parameters with Ang-2 in subjects with AITD and healthy controls. A significant positive correlation of Ang-2 with oxidative stress parameters indicates a close connection between redox imbalance and angiogenesis. In subjects with HT, a positive relationship of Ang-2 with H_2_O_2_ and NO was recorded, while in GD, Ang-2 significantly positively correlated with O_2_^−^ and TBARS. At the same time, in subjects with GD, a negative correlation of Ang-2 with the activities of antioxidant enzymes (SOD and CAT) was observed.

**Figure 9:**
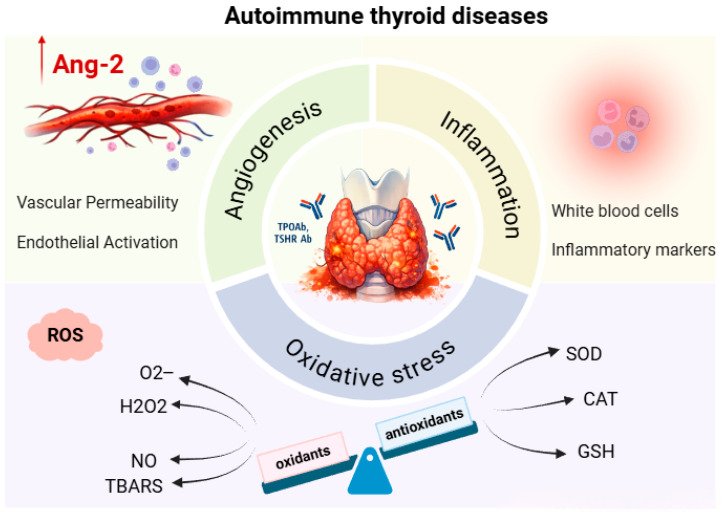
Angiogenesis, inflammation, and oxidative stress: interrelationships in autoimmune thyroid diseases (Created in BioRender. Djordjevic Milanovic, J. (2026) https://BioRender.com/83tbj0p, accessed on 29 January 2026).

Taken together, our results strongly support the concept of a functional association of angiogenesis, inflammation, and oxidative stress in the pathophysiology of AITD. The chronic inflammation characteristic of these disorders promotes increased production of ROS, which leads to redox imbalance and depletion of antioxidant defenses [33]. Such a pro-oxidant environment may further activate endothelial cells and angiogenic signaling pathways, including the Ang-2/Tie-2 axis, thereby enhancing vascular permeability and recruitment of inflammatory cells to the thyroid tissue [34]. At the same time, Ang-2 acts as an important modulator of inflammation, increasing the sensitivity of the endothelium to pro-inflammatory cytokines, thereby establishing a vicious circle in which inflammation, oxidative stress, and angiogenesis reinforce each other and contribute to the progression of autoimmune damage to the thyroid gland [15,34] (Figure 9).

## 4. Materials and Methods

### 4.1. Study Design and Study Population

The research was conducted in the form of a cross-sectional clinical observational study that included 183 subjects of both sexes with AITD examined in the Department of Nuclear Medicine of the University Clinical Center Kragujevac. Subjects with AITD were divided into two groups, namely: subjects with a diagnosis of HT and subjects with a diagnosis of GD. The group of subjects with HT included 97 newly diagnosed subjects with elevated concentrations of thyroid-stimulating hormone (TSH), normal/low concentrations of fT4, positive TPO-Ab and/or antibodies to thyroglobulin (Tg-Ab), and in whom replacement therapy has not yet been introduced. The group of subjects with GD included 86 subjects with low concentrations of TSH, normal/high concentrations of fT4, and positive antibodies to TSHR-Ab before the introduction of thyro-suppressive therapy. A control group consisting of 76 healthy subjects without a prior diagnosis of acute or chronic inflammatory disease, autoimmune disease, or malignant disease was analyzed in parallel.

The study used a consecutive sampling method, including respondents who met the appropriate criteria. The inclusion criteria were as follows: patient age over 18 years, confirmed diagnosis of autoimmune thyroid disease, and signed informed consent for participation in the study. The study did not include participants younger than 18 years of age. Additionally, individuals with previously diagnosed acute infections (up to one month before inclusion in the study) and those with previously diagnosed and/or treated autoimmune and chronic inflammatory diseases or malignant diseases, as well as pregnant women, were excluded from the study.

This study was conducted in accordance with the Declaration of Helsinki and was approved by the Ethics Committee of the University Clinical Center Kragujevac (number 01/23-269). All respondents were familiar with the research procedure before the start of the study and gave their informed consent to participate in the study.

### 4.2. Data Collection

Peripheral blood samples for the analysis of Ang-2, oxidative stress, antioxidant protection, thyroid function, hematological, biochemical, and inflammatory parameters were collected in the morning hours (between 8 and 10 am), after overnight fasting and after obtaining consent to participate in the study. Thyroid function, hematological, biochemical, and inflammatory parameters were determined immediately after sampling. In accordance with the protocol, samples intended for the analysis of oxidative stress and antioxidant protection parameters were stored at −80 °C, while samples for the determination of Ang-2 were stored at ≤−20 °C until analysis. Repeated freeze–thaw cycles were avoided by analyzing all samples for each parameter within one cycle, thereby minimizing potential variability due to storage.

Determination of parameters of thyroid status was performed in the Department of Nuclear Medicine; hematological, biochemical, and inflammation parameters were analyzed in the Department of Laboratory Medicine of the University Clinical Center Kragujevac; the concentration of Ang-2 was determined in the Laboratory of the Department of Allergology and Immunology of the Institute of Public Health in Kragujevac; and the parameters of oxidative stress and antioxidant protection were evaluated at the Center of Excellence for Research on Redox Balance in Cardiovascular and Metabolic Disorders, Faculty of Medical Sciences, University of Kragujevac.

### 4.3. Determination of Angiopoietin-2 Concentration

The concentration of Ang-2 in the serum of the subjects was determined using a commercial Human Angiopoietin-2 kit (USA R&D Systems, Inc., catalog number DANG20, Minneapolis, MN, USA), using the Enzyme-Linked Immuno-Sorbent Assay (ELISA) method, according to the manufacturer’s instructions. The sensitivity of the assay (limit of detection) ranged from 1.20 to 21.3 pg/mL, with an average minimum detection dose of 8.29 pg/mL. The intra-assay coefficients of variation (CV) ranged from 4.2% to 6.9%, and the inter-assay CV ranged from 7.4% to 10.4%. Linearity was assessed by serial dilution of serum with calibrator diluent, and the following linearity ranges were obtained: 87–103% at a 1:2 dilution; 86–112% at 1:4 dilution; and 85–114% at 1:8 dilution. The reference range (1065–8907 pg/mL) was provided by the manufacturer and was determined based on measurements in healthy volunteers [35].

### 4.4. Determination of Redox Status

#### 4.4.1. Blood Sampling

Whole blood samples were collected by venipuncture using commercially available vacutainers with 3.2% sodium citrate. Plasma and erythrocytes were separated by centrifugation for 10 min at 3000 rpm at room temperature. After centrifugation, plasma was stored at −80 °C until oxidative stress biomarkers were measured, while erythrocytes were washed three times with saline. After the third wash, 1 mL of erythrocytes was dissolved in 3 mL of distilled water, and then stored at −80 °C until antioxidant activity was measured.

#### 4.4.2. Determination of Oxidative Stress Parameters

O_2_^−^ levels were assessed using the Nitro Blue Tetrazolium (NBT) assay. Briefly, 50 µL of plasma was combined with 950 µL of washing solution in test tubes. The prepared samples were incubated at room temperature for 10–15 min. Immediately after initiating the reaction, the absorbance of the mixture was measured and recorded as the initial reading (E1). During a period of 5 min, every 60 s, stirring was performed with a plastic stick, after which the excitation was recorded. The last excitation was marked as E2. An equivalent volume of distilled water served as a blank [36,37]. The concentration of O_2_^−^ is obtained using the equation:

ΔEp = E2p − E1p (for plasma samples), ΔEbp = E2bp − E1bp (for blank probe), ΔE = ΔEp − ΔEbp, nmol O_2_^−^ = ΔE/0.015 × 1/0.05 (1)

The measurement was performed at the wavelength of maximum absorption λmax = 550 nm, while the results were expressed as nmol/mL.

H_2_O_2_ concentration was determined using a method based on the peroxidase-catalyzed oxidation of phenol red. In this assay, 200 µL of plasma was mixed with 800 µL of freshly prepared phenol red solution (PRS) and 10 µL of horse radish peroxidase (HRPO). Following incubation at room temperature for 10 min, absorbance was recorded at λ = 610 nm. Distilled water of the same volume was used as the blank control [36,38].

Since NO decomposes rapidly, forming an equal amount of nitrite products, the NO concentration was estimated by indirect measurement of the nitrite concentration (NO_2_^−^). In the spectrophotometric method for biochemical determination of nitrite, Griess reagent was used, which forms a diazo complex with nitrites in a purple color. For this assay, 1 µL of plasma was combined with 250 µL of freshly prepared Griess reagent and 125 µL of ammonium buffer in test tubes. The reaction mixtures were allowed to stand at room temperature for 15 min, after which absorbance was measured at λ = 550 nm. A corresponding volume of distilled water served as the blank control [36,39].

The lipid peroxidation index in plasma samples was determined indirectly by measuring the reaction products of TBARS. TBARS values in plasma samples were determined spectrophotometrically. A total of 800 µL of plasma was mixed with 200 µL of 1% thiobarbituric acid (TBA) prepared in 0.05% sodium hydroxide. The mixtures were heated in a water bath at 100 °C for 15 min, then allowed to cool at room temperature for 10 min. Absorbance was measured at λ = 530 nm, and the results were expressed in μmol/mL. An equal volume of distilled water was used as the blank [36,40].

#### 4.4.3. Determination of Antioxidant Protection Parameters

SOD activity was measured using the Beutler epinephrine method. In brief, 100 µL of erythrocyte lysate was mixed with 1000 µL of carbonate buffer in test tubes. After briefly vortexing, 100 µL of epinephrine was added to initiate the reaction. Absorbance was recorded at λ = 470 nm, and SOD activity was expressed as units per gram of hemoglobin (U/g Hb). A matching volume of distilled water was used as the blank [36,41].

CAT activity was determined following the Aebi method, which monitors the spectrophotometric decomposition of H_2_O_2_ by catalase. In this assay, 50 µL of CAT buffer, 100 µL of prepared lysate, and 1000 µL of H_2_O_2_ were combined in test tubes. Absorbance was measured six consecutive times at λ = 360 nm. A corresponding volume of distilled water was used as the blank [36,42].

GSH levels were measured using the Beutler method, based on the oxidation of GSH by 5,5’-dithio-bis-6,2-nitrobenzoic acid (DTNB). Briefly, 50 µL of lysate was mixed with 200 µL of 0.1% EDTA and 385 µL of precipitation buffer, then placed on ice for 15 min and centrifuged for 15 min. From the resulting extract, 300 µL was combined with 750 µL of dibasic sodium phosphate and 100 µL of DTNB. Following a 10-min incubation, absorbance was recorded at λ = 420 nm. An equal volume of distilled water was used as the blank [36,43].

### 4.5. Determination of Thyroid Function Parameters

The concentrations of fT3 and fT4 were measured with radioimmunoassay kits (DIAsource ImmunoAssays S.A., Belgium, Germany) and the concentration of Tg was measured with an immunoradiometric kit (DIAsource ImmunoAssays S.A., Belgium, Germany) [44]. The concentration of TSH was determined with an immunoradiometric test (Company INEP, Beograd (Zemun), Serbia) [45], while the titers of Tg-Ab, TPO-Ab, and TSHR-Ab were determined with radioimmunoassay tests (Immunotech, Prague, Czech Republic) [46]. Some detailed characteristics of the assays, according to the manufacturer’s instructions, are given in Table 4. The ranges of reference values according to the manufacturer for the listed parameters are as follows: fT3 2.50–5.80 pmol/L, fT4 11.50–23.0 pmol/L, Tg 0–50 ng/mL, TSH 0.3–5.5 mIU/L, Tg-Ab 0–30 IU/mL, TPO-Ab 0–12 IU/mL, and TSH R-Ab 0-1 [44,45,46].

### 4.6. Determination of Hematological, Biochemical, and Inflammatory Parameters

Hematological parameters (hemoglobin concentration, hematocrit, iron, erythrocyte count, leukocyte count and leukocyte formula, platelet count, platelet indices) were examined automatically using a DxH 800 Hematology Analyzer (Beckman Coulter, Inc., Brea, CA, USA).

The concentration of biochemical parameters (urea, creatinine, uric acid, albumin, vitamin D concentration) was determined on a Beckman Coulter AU5800 analyzer, using the laboratory method of spectrophotometry.

To assess the presence of inflammation, serum concentrations of CRP were analyzed using the Beckman Coulter CRP Latex reagent on a Beckman Coulter AU5800 analyzer, as well as IL-6 concentrations measured using commercially available enzymatic reagents adapted to the Cobas e 411 chemical analyzer (Roche diagnostics GmbH, Mannheim, Germany). Simultaneously, inflammatory status was evaluated using several indices: the neutrophil-to-lymphocyte ratio (NLR) and platelet-to-lymphocyte ratio (PLR); the systemic inflammatory response index (SIRI), calculated as the product of neutrophil and monocyte counts divided by lymphocyte count; the systemic immune-inflammation index (SII), defined as the product of neutrophils and platelets divided by lymphocytes; and the CRP to albumin ratio (CRP/alb) [47].

### 4.7. Statistical Analysis

The data were analyzed using SPSS version 22.0. Continuous variables are reported as mean ± standard deviation, while categorical variables as proportions of respondents. Before applying statistical tests, to check for normality of the data, the Kolmogorov–Smirnov and Shapiro–Wilk tests were applied. Depending on whether the data were normally distributed or no, group comparisons of continuous variables were conducted using either one-way ANOVA or the non-parametric Kruskal–Wallis test. In one-way ANOVA, Levene’s test was performed to assess the equality of variances between groups. Subsequent comparisons after the one-way ANOVA were performed using the Tukey HSD test, while after the Kruskal–Wallis test, pairwise comparisons (Mann–Whitney U test) were performed with Bonferroni correction. The chi-square (χ^2^) test was used to assess differences in the frequency of categorical variables. Linear correlation tests were used to analyze the relationship between dependent and independent variables, determining the Pearson correlation coefficient for normally distributed data, or the Spearman rank correlation coefficient for non-normally distributed data. Values of *p* ≤ 0.05 were considered statistically significant.

The heatmap was created using https://www.bioinformatics.com.cn/en (accessed on 25 December 2025).

## 5. Conclusions

Based on the obtained results, it can be concluded that patients with AITD show pronounced disorders in angiogenic, inflammatory, and redox mechanisms, whereby clear differences between HT and GD can be observed. Elevated concentrations of Ang-2, especially in patients with GD, together with its significant association with inflammatory and pro-oxidative parameters, indicate its important role in the pathophysiology of these disorders. At the same time, the reduced activity of antioxidant protection enzymes, especially in patients with HT, confirms the presence of redox imbalance as one of the key mechanisms of autoimmune dysfunction of the thyroid gland. In conclusion, our findings suggest that Ang-2 may represent a potential biomarker of disease activity and a link between inflammation, oxidative stress, and angiogenesis in AITD, opening opportunities for new therapeutic and diagnostic considerations.

### Limitations

A limitation of the study is its retrospective design without a prospective component. Additional prospective studies are needed to confirm the relevance of our results and their applicability in clinical practice.

## Figures and Tables

**Table 1:** Thyroid function parameters of the study population.

Parameter	Subjects with Hashimoto’s Thyroiditis		Subjects with Graves’ Disease		Control Subjects		Significance
	Mean ± SD	Min–Max	Mean ± SD	Min–Max	Mean ± SD	Min–Max	
fT3 (pmol/L)	4.43 ± 1.09	2.80–6.80	6.12 ± 3.80	2.30–20.90	5.05 ± 0.88	3.10–7.08	**0.024**
fT4 (pmol/L)	11.11 ± 3.91	3.28–19.8	23.32 ± 9.06	11.50–46.30	10.72 ± 2.27	6.39–21.0	**<0.001**
TSH (mIU/L)	7.40 ± 4.42	0.4–17.0	0.18 ± 0.39	0.01–3.21	2.10 ± 1.16	0.3–5.59	**<0.001**
TPO-Ab (IU/mL)	227.07 ± 81.32	0.30–1604	248.95 ± 95.03	0.40–1500	1.42 ± 2.44	0.10–11.70	**<0.001**
Tg (ng/mL)	11.35 ± 15.43	0.00–84.30	28.38 ± 41.61	0.10–156	10.03 ± 12.58	0.10–77.80	0.669
Tg-Ab (IU/mL)	135.63 ± 196.76	0.10–2000	61.79 ± 12.22	0.10–455	1.04 ± 2.40	0.00–16.10	**<0.001**
TSHR-Ab (U/L)	1.04 ± 2.19	0.10–9.40	10.72 ± 7.66	0.10–32.20	0.22 ± 0.21	0.10–0.90	**<0.001**
fT3/fT4 (ratio)	0.55 ± 0.32	0.21–1.67	0.31 ± 0.21	0.09–1.21	0.48 ± 0.10	0.28–0.77	**<0.001**

Abbreviations: fT3, free triiodothyronine; fT4, free thyroxine; TSH, thyroid-stimulating hormone; TPO-Ab, thyroid peroxidase antibodies; Tg, thyroglobulin; Tg-Ab, thyroglobulin antibodies; TSHR-Ab, Thyroid-Stimulating Hormone Receptor Antibodies.

**Table 2:** Hematological and biochemical parameters in the study population.

Parameter	Subjects with Hashimoto’s Thyroiditis		Subjects with Graves’ Disease		Control Subjects		Significance
	Mean ± SD	Min–Max	Mean ± SD	Min–Max	Mean ± SD	Min–Max	
Erythrocytes (×10^12^/L)	4.53 ± 0.53	2.95–5.75	4.54 ± 0.71	2.37–5.92	4.04 ± 0.75	3.21–5.37	0.537
Hemoglobin (g/L)	130.42 ± 24.76	88.0–173.0	134.97 ± 19.12	103.0–175.0	134.63 ± 18.99	84.0–171.0	0.866
Hematocrit (L/L)	0.39 ± 0.09	0.27–0.83	0.40 ± 0.05	0.29–0.53	0.40 ± 0.05	0.25–0.51	0.897
Leukocyte (×10^9^/L)	9.29 ± 3.62	4.90–16.6	9.55 ± 1.60	4.26–17.10	6.07 ± 1.38	3.70–9.52	** <0.001 **
Neutrophils (×10^9^/L)	5.43 ± 1.47	2.20–10.0	5.29 ± 1.73	2.45–9.70	3.55 ± 0.82	1.90–5.70	** <0.001 **
Monocytes (×10^9^/L)	0.94 ± 1.42	0.20–6.80	0.81 ± 0.83	0.30–4.60	0.54 ± 0.18	0.04–1.31	0.128
Lymphocytes (×10^9^/L)	3.04 ± 1.48	1.30–8.90	2.73 ± 1.59	1.00–9.00	2.22 ± 0.94	0.90–5.60	** 0.008 **
Platelets (×10^9^/L)	369.10 ± 56.06	212.0–474.0	351.97 ± 54.40	225.0–468.0	278.02 ± 73.14	117.0–484.0	** <0.001 **
MPV (fl)	9.87 ± 0.98	7.70–12.90	9.59 ± 0.92	7.10–11.50	8.5 ± 1.02	7.0–11.30	** <0.001 **
PDW (ratio)	16.69 ± 1.97	11.10–19.90	15.79 ± 1.74	8.70–18.70	13.80 ± 2.33	9.60–17.20	** <0.001 **
Iron (umol/L)	17.12 ± 5.15	7.40–26.10	18.02 ± 3.75	13.40–25.30	12.26 ± 1.23	5.60–22.60	** 0.004 **
Urea (mmol/L)	5.41 ± 2.75	1.50–20.20	6.90 ± 4.03	1.84–16.60	4.70 ± 1.31	1.80–7.60	** 0.030 **
Creatinine (umol/L)	74.75 ± 19.28	43.0–144.0	72.70 ± 20.33	35.0–142.0	66.85 ± 15.82	38.0–106.0	0.700
Uric acid (umol/L)	278.60 ± 69.05	135.0–390.0	387.59 ± 50.71	304–515.00	296.51 ± 90.68	157.0–503.0	** <0.001 **
Albumin (g/L)	42.84 ± 4.25	35.0–51.0	41.65 ± 4.88	32.0–50.0	44.16 ± 3.86	34.0–50.0	0.160
Vitamin D (ng/mL)	23.83 ± 9.34	4.40–45.90	24.03 ± 9.70	7.50–48.98	28.98 ± 6.27	20.32–52.14	** 0.002 **

Abbreviations: MPV, mean platelet volume; PDW, platelet distribution width.

**Table 3:** Inflammation parameters in the study population.

Parameter	Subjects with Hashimoto’s Thyroiditis		Subjects with Graves’ Disease		Control Subjects		Significance
	Mean ± SD	Min–Max	Mean ± SD	Min–Max	Mean ± SD	Min–Max	
CRP (mg/L)	7.52 ± 1.25	0.70–49.57	10.76 ± 2.51	1.30–60.20	1.60 ± 0.92	0.10–6.46	** <0.001 **
IL6 (pg/mL)	7.67 ± 3.86	2.49–18.97	11.17 ± 4.95	3.21–19.60	3.02 ± 2.05	1.50–9.60	** <0.001 **
NLR (ratio)	2.01 ± 0.81	0.65–4.55	2.23 ± 1.28	0.65–6.70	1.87 ± 0.75	0.61–5.44	0.270
PLR (ratio)	38.75 ± 5.97	38.09–316.0	46.92 ± 6.28	40.0–367.0	40.75 ± 5.41	38.57–234.78	0.773
SIRI (ratio)	1.60 ± 0.86	0.29–11.96	1.58 ± 0.91	0.49–4.49	1.01 ± 0.57	0.08–2.85	0.054
SII (ratio)	91.57 ± 5.77	220.92–1738.0	96.65 ± 5.02	227–2458	50.22 ± 6.07	131.14–914.67	** <0.001 **
CRP/alb (ratio)	0.15 ± 0.09	0.01–1.42	0.29 ± 0.06	0.03–1.04	0.03 ± 0.02	0.01–0.88	** <0.001 **

Abbreviations: CRP, C reactive protein; IL6, interleukin 6; NLR, neutrophil-to-lymphocyte ratio; PLR, platelet-to-lymphocyte ratio; SIRI, Systemic Inflammation Response Index; SII, Systemic Immune-inflammation Index; CRP/alb, C-Reactive Protein/Albumin Ratio.

**Table 4:** Characteristics of RIA and IRMA according to the manufacturers [44,45,46].

Parameters	fT3	fT4	TSH	Tg	Tg-Ab	TPO-Ab	TSH R-Ab
Principle of the assay	RIA	RIA	IRMA	IRMA	RIA	RIA	RIA
Sensitivity of the assay (detection limit)	1.0 pmol/L	2.34 pmol/L	0.04 mIU/L	0.42 ng/mL	19.7 IU/mL	2.81 IU/mL	0.33 U/L
Measuring range	0.5–40 pmol/L	0.4–75 pmol/L	0.15–50 mIU/L	0.75–600 ng/mL	2.02–3000 IU/mL	2.81–2500 IU/mL	0.1–40 U/L
Intra- assay reproducibility	6.4%	10.29%	2.1%	1.6%	10.4%	11.7%	10%
Inter-assay reproducibility	5.5%	7.58%	7.1%	2.2%	10.2%	15%	15%

## Data Availability

The original contributions presented in this study are included in the article. Further inquiries can be directed to the corresponding author.
